# Identification of alterations in macrophage activation associated with disease activity in systemic lupus erythematosus

**DOI:** 10.1371/journal.pone.0208132

**Published:** 2018-12-18

**Authors:** Adam C. Labonte, Brian Kegerreis, Nicholas S. Geraci, Prathyusha Bachali, Sushma Madamanchi, Robert Robl, Michelle D. Catalina, Peter E. Lipsky, Amrie C. Grammer

**Affiliations:** 1 AMPEL BioSolutions LLC, Charlottesville, Virginia, United States of America; 2 RILITE Research Institute, Charlottesville, Virginia, United States of America; Instituto Nacional de Ciencias Medicas y Nutricion Salvador Zubiran, MEXICO

## Abstract

Systemic lupus erythematosus (SLE) is characterized by abnormalities in B cell and T cell function, but the role of disturbances in the activation status of macrophages (Mϕ) has not been well described in human patients. To address this, gene expression profiles from isolated lymphoid and myeloid populations were analyzed to identify differentially expressed (DE) genes between healthy controls and patients with either inactive or active SLE. While hundreds of DE genes were identified in B and T cells of active SLE patients, there were no DE genes found in B or T cells from patients with inactive SLE compared to healthy controls. In contrast, large numbers of DE genes were found in myeloid cells (MC) from both active and inactive SLE patients. Among the DE genes were several known to play roles in Mϕ activation and polarization, including the M1 genes STAT1 and SOCS3 and the M2 genes STAT3, STAT6, and CD163. M1-associated genes were far more frequent in data sets from active versus inactive SLE patients. To characterize the relationship between Mϕ activation and disease activity in greater detail, weighted gene co-expression network analysis (WGCNA) was used to identify modules of genes associated with clinical activity in SLE patients. Among these were disease activity-correlated modules containing activation signatures of predominantly M1-associated genes. No disease activity-correlated modules were enriched in M2-associated genes. Pathway and upstream regulator analysis of DE genes from both active and inactive SLE MC were cross-referenced with high-scoring hits from the drug discovery Library of Integrated Network-based Cellular Signatures (LINCS) to identify new strategies to treat both stages of SLE. A machine learning approach employing MC gene modules and a generalized linear model was able to predict the disease activity status in unrelated gene expression data sets. In summary, altered MC gene expression is characteristic of both active and inactive SLE. However, disease activity is associated with an alteration in the activation of MC, with a bias toward the M1 proinflammatory phenotype. These data suggest that while hyperactivity of B cells and T cells is associated with active SLE, MC potentially direct flare-ups and remission by altering their activation status toward the M1 state.

## Introduction

SLE is typically characterized by B cell hyperactivity and autoantibody formation, promoted by T cell dysregulation.[1] The role of MC in SLE, however, remains poorly understood despite their considerable influence on adaptive immunity. Mϕ and dendritic cells (DCs) are phagocytic professional antigen presenting cells (APC) of myeloid lineage that are integral to the propagation and orchestration of immune responses. Although DCs are the main myeloid cell (MC) population responsible for antigen presentation, phagocytosed antigens are also processed by Mϕ and presented on the Mϕ surface by MHC-I and–II molecules to activate both B cells [2] and T cells [3–5].

Bone marrow (BM)-derived Mϕ originate from hematopoietic stem cells (HSC) that differentiate into common myeloid progenitor (CMP) cells and subsequently into monocytes.[6] Upon activation, patrolling monocytes further differentiate into Mϕ to address the injury or infection they have detected. DCs also originate from myeloid progenitors, specifically from the common DC progenitor (CDP) which develops from the CMP along with monocytes. The CDP gives rise to both plasmacytoid DCs (pDC) and pre-DCs, which give rise to classical DCs (cDC). [7] pDCs, which are identified by expression of B220, Siglec-H, and Bst2, are less phagocytic and less efficient APC and instead are responsible for producing large amounts of type I interferon to combat viral infections. [8,9]

Mϕ express a large collection of surface receptors to monitor their local microenvironment that allows them to act as sentinels for markers of infection or injury.[10] Engagement of these receptors by cell debris, viral or bacterial byproducts, cytokine and chemokine signals, and other factors activates Mϕ and allows them to modify their phenotype and function rapidly and contribute to host defense. [11–13] Mϕ combat infectious disease both through intracellular destruction of phagocytosed pathogens and via production of various antimicrobial peptides, reactive oxygen intermediates, and nitric oxide. [14,15] Other innate functions of activated Mϕ include wound repair and tissue remodeling [16], and proinflammatory Mϕ are thought to eliminate tumor cells in the early stages of cancer. [17] As early responders at sites of inflammation and infection, Mϕ also shape the early adaptive immune response by reacting to changes in the microenvironment and secreting various chemokines and cytokines to recruit other immune cells. [18]

Specific stimulating factors and signals cause Mϕ to undergo extreme changes in transcriptional regulation and assume a specific activation state ranging from highly proinflammatory to anti-inflammatory in a process called Mϕ polarization. [19–21] Each polarization state or subset expresses a particular profile of surface receptors, cytokines, chemokines, and secreted effector molecules that dictates its functional effect on inflammation, immune cell recruitment and activation (or suppression), and tissue remodeling. [22,23] Named in accordance with the Th1/Th2 paradigm of immune responses, the M1 and M2 polarization states represent canonical proinflammatory and anti-inflammatory Mϕ functional states, respectively, and indeed, produce cytokines and chemokines that correspond to Th1 and Th2 response induction.[24] The whole of Mϕ polarization, however, represents a spectrum of overlapping phenotypic states between M1 and M2 Mϕ, and several other subsets between these extremes have been defined in various disease models.[25–28]

There has been growing appreciation for the contribution of Mϕ polarization to both disease progression and resolution. Alteration of the M1/M2 Mϕ balance has been shown to have crucial roles in bacterial and viral infections, and many pathogens have evolved escape mechanisms that manipulate Mϕ polarization to enhance their survival and spread.[29,30] M1 and M2 Mϕ also influence local inflammation, the dysregulation of which is central to the pathology of diseases with inflammatory components, including type 1 diabetes, obesity, non-alcoholic steatohepatitis, atherosclerosis, and Crohn’s disease.[29–32] Recent studies have begun to explore the contribution of Mϕ to SLE-like disease pathogenesis in mice, but a lack of human studies has hindered the investigation of activated Mϕ as potential contributors to molecular pathology and as therapeutic targets.[33] To address this, here we employ a bioinformatics-based approach to examine the myeloid-derived genomic signatures that define both active and inactive SLE in human patients and to identify promising candidates empirically for drug intervention.

## Methods

### Selection, QC, and normalization of raw data files

Raw data files for human peripheral myeloid cells purified from SLE patients and healthy controls (HC) were obtained from the publicly accessible Gene Expression Omnibus (GEO) repository (CD33^+^ cells [GSE10325; 10HC, 7 active SLE] and CD14^+^ cells [GSE38351; 12HC, 8 active SLE, 5 inactive SLE]). [34,35] SLE patients with an SLE Disease Activity Index (SLEDAI) score less than six were defined as having inactive disease, whereas those with a SLEDAI score of 6 or greater were defined as having active disease. Raw data files for T and B cells isolated from SLE patients or HCs were obtained from GEO to be used for later comparative analyses (GSE10325 [CD4^+^ T cells, CD19^+^ B cells], GSE51997 [active CD4^+^ T cells], and GSE4588 [active CD19^+^ B cells]). [34,36] Accession numbers, descriptions, and cell types for all datasets used are summarized in S1 Table.

Processing of raw data files, obtained for each respective study on GEO, was conducted with Bioconductor packages GEOquery, affy, affycoretools, and simpleaffy in R. Raw array data were inspected for visual artifacts or poor RNA hybridization using Affymetrix QC plots. Datasets that passed quality control measures were normalized using the GCRMA method (guanine cytosine robust multiarray averaging), and transformed to obtain log2 intensity values, which were formatted into R expression set objects (E-sets). Principal component analysis (PCA) plots were generated for all cell types in each experiment to inspect for outlier samples, admixed disease cohorts, and batch effects visually.

Raw microarray data were annotated using chip definition files (CDF) appropriate to the microarray product from Affymetrix. In order to identify additional genes unrecognized by Affymetrix CDFs, the same data were subsequently processed and annotated using custom BrainArray CDF version 19.[37] Probe sets lacking annotations by the Affymetrix CDF were interrogated for BrainArray definitions. Any probes that were annotated by Affymetrix CDF but also were incorporated in BrainArray probe sets identifying alternative genes were excluded. For Affymetrix HGU133A platform microarrays, a total of 12,504 genes were identified by Affymetrix CDF. Of these, 11,825 were also identified by BrainArray and an additional 354 genes were identified by BrainArray alone, whereas 143 Affymetrix probe sets were excluded.

### Differential gene expression analysis

The annotated E-sets were filtered to remove probes with very low intensity values via visual operator selection of thresholds set at the trough of low intensity histogram frequencies, post-normalization. Any probes that lacked gene annotation data were also discarded. GCRMA normalized expression values were variance corrected using local empirical Bayesian shrinkage before calculation of DE using the ebayes function in the Bioconductor LIMMA package. Resulting p-values were adjusted for multiple hypothesis testing using the Benjamini-Hochberg correction which reports a false discovery rate (FDR). Probe sets within each study were filtered to retain differentially expressed (DE) probes with an *a priori* FDR < 0.2 which were considered statistically significant. This FDR cutoff was employed with the understanding that additional false positive probes might be included in the analysis but that fewer false negative probes would be inappropriately excluded. Since additional analyses that did not involve an estimate of FDR were included to confirm the results and exclude the contributions of false positives, we were more concerned about excluding apparent false negatives from the analysis. This list was further filtered to retain only the most significant probe per gene in order to remove duplicate probes.

### Weighted gene co-expression network analysis (WGCNA)

Log2 normalized microarray expression values were used as input to WGCNA (v1.60) to conduct an unsupervised clustering analysis, resulting in co-expression modules (groups of densely interconnected genes) which correspond to comparably regulated biological pathways.[38] For each experiment, an approximately scale-free topology matrix (TOM) was first calculated to encode the network strength between probes. Probes were clustered into WGCNA modules based on TOM distances. Resultant dendrograms of correlation networks were trimmed to isolate individual modular groups of probes, labeled using semi-random color assignments, based on a detection cut height of 1 and a merging cut height of 0.2, with the additional use of a partitioning around medoids function. Final membership of probes representing the same gene into modules was based on selection of the greatest within-module correlation with module eigengene (ME) values. Expression profiles of genes within modules were summarized by the ME, the module’s first principal component. MEs act as characteristic expression values for their respective modules and can be correlated with sample traits such as cell type, cohort (healthy control or SLE), or serological measurements. This was done by Pearson correlation for continuous traits and by point-biserial correlation for dichotomous traits. The correlation coefficient of each gene in a module with the module eigengene (kME), a metric for module membership, was used to determine the association of individual genes with the expression of the module as a whole. The mean kME of all genes in a module was taken as a metric of overall module quality. If the genes in a module have low kMEs, it is indicative that a few highly variable genes have dominated the eigengene calculation. Modules with mean kMEs close to 1 were considered to be high-quality, and modules with mean kMEs close to zero were considered to be low-quality. When analyzing multiple data sets, the grand mean was the mean of the mean kMEs for each data set.

### Functional gene characterization and pathway identification

The Biologically Informed Gene Clustering (BIG-C)[39] tool characterizes genes into functional groups utilizing publicly available information from online tools and databases including UniProtKB/Swiss-Prot, GO Terms, KEGG pathways, NCBI PubMed, and the Interactome. DE genes were assigned into functional groups using BIG-C and signaling molecules and transcription factors upstream of DE genes were identified using IPA Upstream Regulator (UR) analysis.[40,41] For each regulator, an activation z-score was calculated strictly from experimentally observed information provided for the downstream targets, and an overlap p-value was calculated through Fischer’s exact test.

### Gene set variation analysis (GSVA)

GSVA (V1.25.4) software package for R/Bioconductor was used as a non-parametric, unsupervised method for estimating the variation of pre-defined gene sets in patient and control samples of microarray expression data sets. The input for the GSVA algorithm was a gene expression matrix of log2 microarray expression values and a collection of pre-defined gene sets or database of pre-exiting gene sets (MSig). Enrichment scores (GSVA scores) were calculated non-parametrically using a Kolmogorov Smirnoff (KS)-like random walk statistic and a negative value for a particular sample and gene set. Significance of functional enrichment was calculated using a chi-squared test and categories with p-values less than 0.05 were considered significantly enriched.

### Network analysis and visualization

Visualization of protein-protein interactions and relationships between genes within datasets was done using the Cytoscape (V3.6.0) software [42] and the stringApp (V1.3.2) plugin application. The Clustermaker2 App (V1.2.1) plugin was used to create clusters of the most related genes within a dataset using a network scoring degree cutoff of 2 and setting a node score cut-off of 0.2, k-Core of 2 and a max depth of 100.

### CIRCOS visualization

CIRCOS (V0.69.3) software was used to visualize datasets.

### Drug target prediction

Queries of the perturbation database from the Broad Institute Library of Integrated Network-Based Cellular Signatures (LINCS) were utilized to predict potentially useful therapeutic compounds and to confirm the dysregulation of upstream target genes in SLE patient MC by assessing signatures of significantly up- and down-regulated genes for input to the lincscloud API (http://data.lincscloud.org.s3.amazonaws.com/index.html). [43] The LINCS L1000 platform was developed using Luminex Flexmap 3D bead technology that contained far greater probe sets than the hgU133 arrays. The LINCS L1000 currently contains representative information relating expression of 978 “landmark genes” that was generated from 25 cell types that were antagonized by drugs and gene over-expression or silencing interventions.

### Prediction of disease activity from WGCNA module enrichment using machine learning

4 whole blood (WB) and 2 peripheral blood mononuclear cell (PBMC) microarray datasets containing gene expression data from lupus patients were obtained from the GEO repository or from collaborators (GSE45291, GSE39088, GSE49454, GSE72747, GSE50772, FDAPBMC3 [S1 File]).[44–49] Raw data was curated and normalized as described previously. In addition, low-intensity probes were filtered, and duplicate probes mapping to the same gene symbol were filtered based on interquartile range. Datasets were batch corrected to account for platform differences using the ComBat R package and merged by matching gene symbols. WGCNA was applied to CD4 T cells (GSE10325), CD19 B cells (GSE10325), CD33 MC (GSE10325), CD14 MC (GSE38351), and low-density granulocytes (LDG) (GSE26975, [50]) to acquire gene modules with significant correlations with or against SLEDAI. GSVA was used to test the merged blood dataset for the presence of these modules as well as lists of genes positively and negatively associated with lupus plasma cells (PC).[51] GSVA scores were used as input to a generalized linear model (GLM) from the glmnet R package to predict disease activity, and receiver operating characteristic (ROC) curves were generated using the pROC R package. Patient-by-patient enrichment of cell types was assessed based on the expected versus observed enrichment of each WGCNA module. Odds ratios (OR) for active disease were calculated according to the following formula:

$$\frac{\left(enriched\;active)*(non-enriched\;inactive\right)}{\left(non-enriched\;active)*(enriched\;inactive\right)}$$

## Results

### Differential expression of MC genes in active and inactive SLE

**Fig 1:**
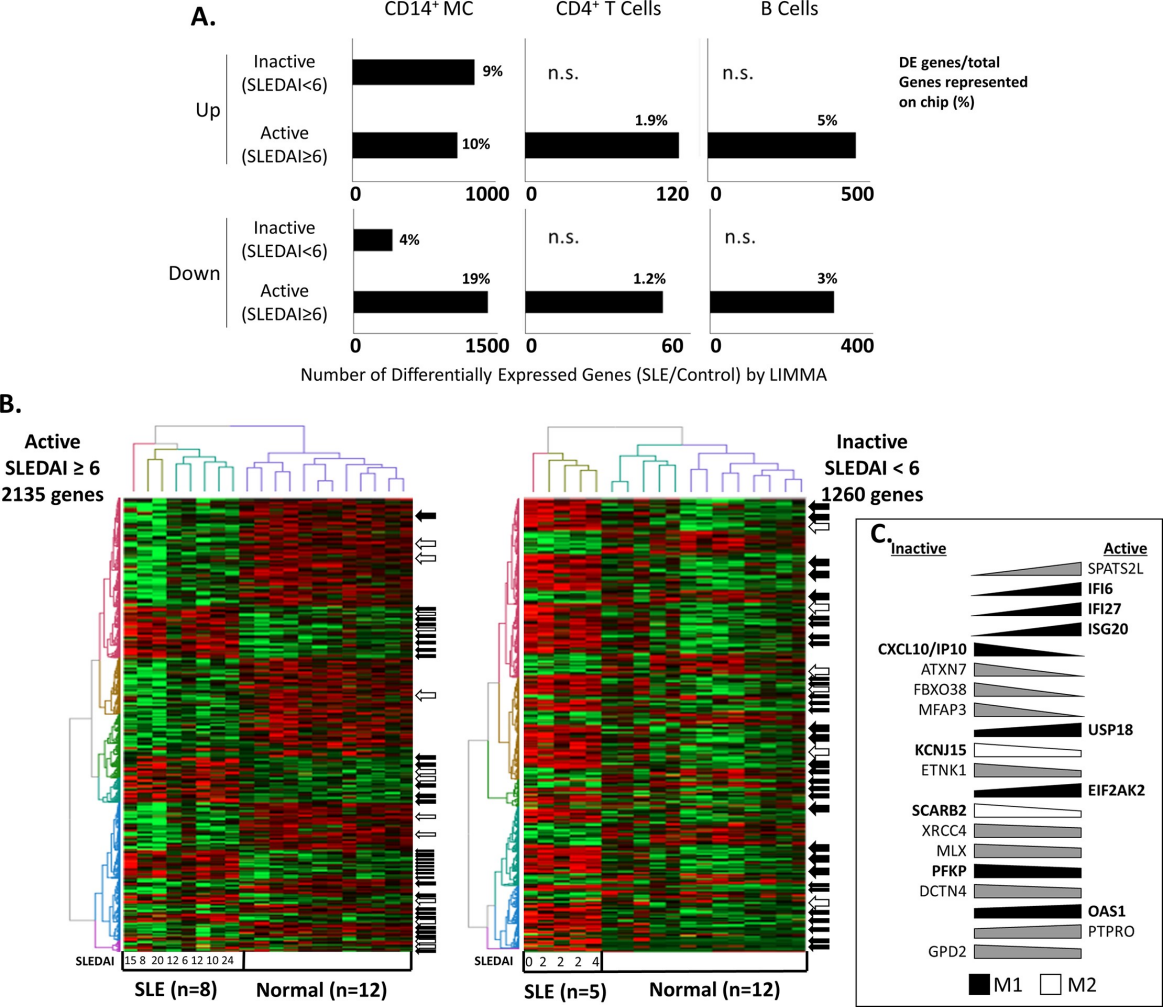
Differential expression of CD14^+^ monocyte genes in active and inactive SLE. (A) Number of differentially expressed (DE) genes detected by LIMMA analysis in MC, CD4^+^ T cells, and B cells isolated from inactive (SLEDAI<6) and active (SLEDAI≥6) SLE patients when compared to healthy donors. n.s.: no genes found to be significantly differentially expressed (FDR<0.2) when compared to healthy controls. (B) Hierarchical clustering of differentially expressed (DE) genes detected by LIMMA analysis in CD14^+^ MC isolated from inactive (SLEDAI<6) and active (SLEDAI≥6) SLE patients when compared to healthy donors. Arrows highlight M1 (black) or M2 (white) polarization genes as reported by Martinez et al.[52] (C) Fold change variation of genes found to be upregulated in both active and inactive SLE MC. Polarization-related genes are shown in bold and M1 genes are represented by a black wedge while M2 genes are represented with a white wedge. Genes not associated with M1 or M2 pathways are represented with a gray wedge.

To assess the contribution of MC to SLE pathogenesis, we analyzed gene expression profiles of CD14^+^ MC from SLE patients with varying levels of disease severity. In order to compare the role of MC in SLE to that of B and T cells, a consensus DE gene signature was generated for each (GSE10325 and GSE51997 for CD19^+^ B cells, CD10325 and CD4588 for CD4^+^ T cells). Large numbers of DE genes were found in MC from both active (2,135) and inactive (1,260) SLE patients (Fig 1A). In contrast, hundreds of statistically significant (FDR<0.2) DE genes were identified in B and CD4 T cells of active SLE patients (760 and 164 genes respectively), whereas there were no significant DE genes found in B or CD4 T cells from patients with inactive SLE compared to healthy controls (Fig 1A).

Hierarchical clustering of DE genes in CD14^+^ MC isolated from inactive and active SLE patients when compared to healthy donors cleanly sorted patient samples by disease cohort (Fig 1B). Although they did not tend to group into discrete clusters, several genes involved in Mϕ activation were observed among the DE genes in both active and inactive patients (S2 Table). Cross-referencing with a list of experimentally determined human Mϕ differentiation and activation genes (Martinez et al., 2006)[52] revealed alterations in the Mϕ activation signature between active and inactive SLE patients: M1-associated genes tended to be upregulated in both active and inactive SLE compared to healthy donors (94% and 97%), while M2-associated genes tended to be more upregulated in inactive SLE patients (86%) than active SLE patients (38%) compared to healthy donors (Fig 1B and S2 Table). As Mϕ activation is known to encompass a spectrum of functional phenotypes controlled by finely-tuned molecular rheostats, we compared the fold change of DE genes that were commonly upregulated in CD14^+^ MC from both active and inactive SLE patients and found that common M1-associated genes (black wedges) were more highly upregulated in active patients whereas common M2-associated genes (white wedges) were more highly upregulated in inactive patients (Fig 1C). A few of these commonly upregulated genes were not associated with either M1 or M2 pathways (gray wedges).

### Functional characterization of DE gene signatures in CD14^+^ MC isolated from SLE patients

**Fig 2:**
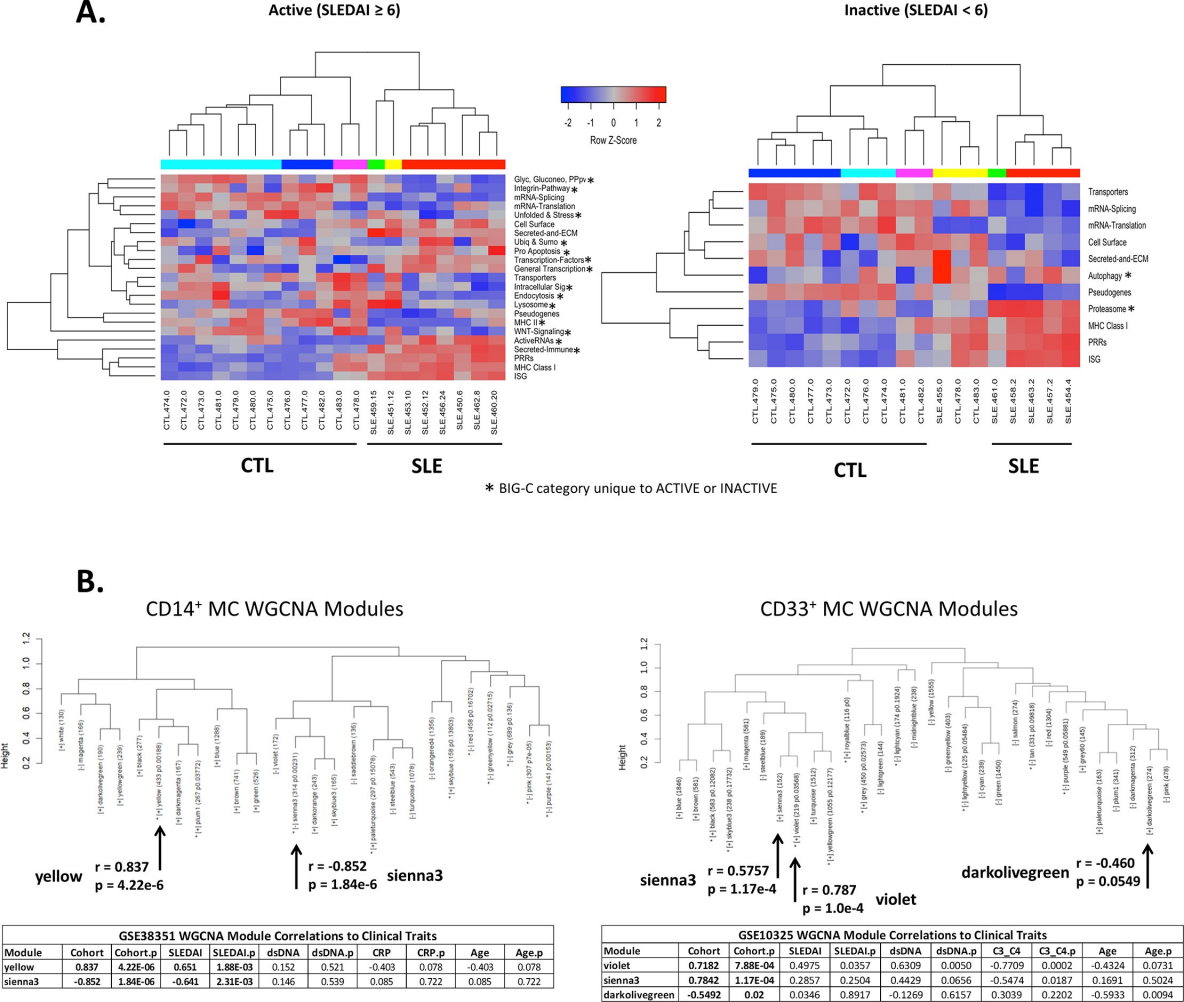
Clustering and module formation of DE gene signatures in CD14^+^ and CD33^+^ MC isolated from SLE Patients. (A) DE genes from active and inactive CD14^+^ MC were analyzed by GSVA to determine pathway enrichment using functional definitions provided from the BIG-C (Biologically Informed Gene Clustering) annotation library. Samples were successfully sorted by disease cohort via this method in both active and inactive MC. Starred BIG-C categories only appeared in the active or inactive analysis, respectively. (B) WGCNA of CD14^+^ and CD33^+^ MC isolated from SLE patients. Dendrograms show hierarchy of modules formed by unsupervised WGCNA clustering of DE genes from CD14^+^ and CD33^+^ MC isolated from active and inactive SLE patients.

We next sought to characterize the potential functional changes represented by the divergent activation signatures in SLE MC. Biologically Informed Gene Clustering (BIG-C) is a functional aggregation tool developed to understand the biological groupings of large lists of genes.[39] Genes are sorted into 54 categories based on their most likely biological function and/or cellular localization determined from information from multiple online tools and databases. The DE genes from active and inactive CD14^+^ MC were analyzed by Gene Set Variation Analysis (GSVA) to determine enrichment of BIG-C functional categories. The active and inactive CD14^+^ MC samples shared a common BIG-C profile generally related to IFN signaling and inflammation, including the MHC class I/II, ISG, immune secreted, transcription, endosomal recycling, immune signaling, and TLR & DAMP categories (Fig 2A). Interestingly, BIG-C categories unique to each cohort (starred) confirm effector function upregulation in MC derived from active SLE (biochem, chromatin, anti-apoptosis down; activeRNAs, secreted & extracellular matrix, immune cell surface, vesicles & endosome up) and a preference for catabolic pathways in MC derived from inactive SLE (cell surface, DNA repair down; ubiquitylation and sumoylation up) (Fig 2A). Additionally, unique enrichment of the MT OX PHOS pathway in MC from inactive SLE mirrors findings that pro-resolving M2 Mϕ predominantly obtain energy from oxidative metabolism.[53,54] A complete list of genes used to determine BIG-C category enrichment can be found in S3 Table.

### MC activation signature genes found in disease-correlated WGCNA modules from active SLE MC

**Fig 3:**
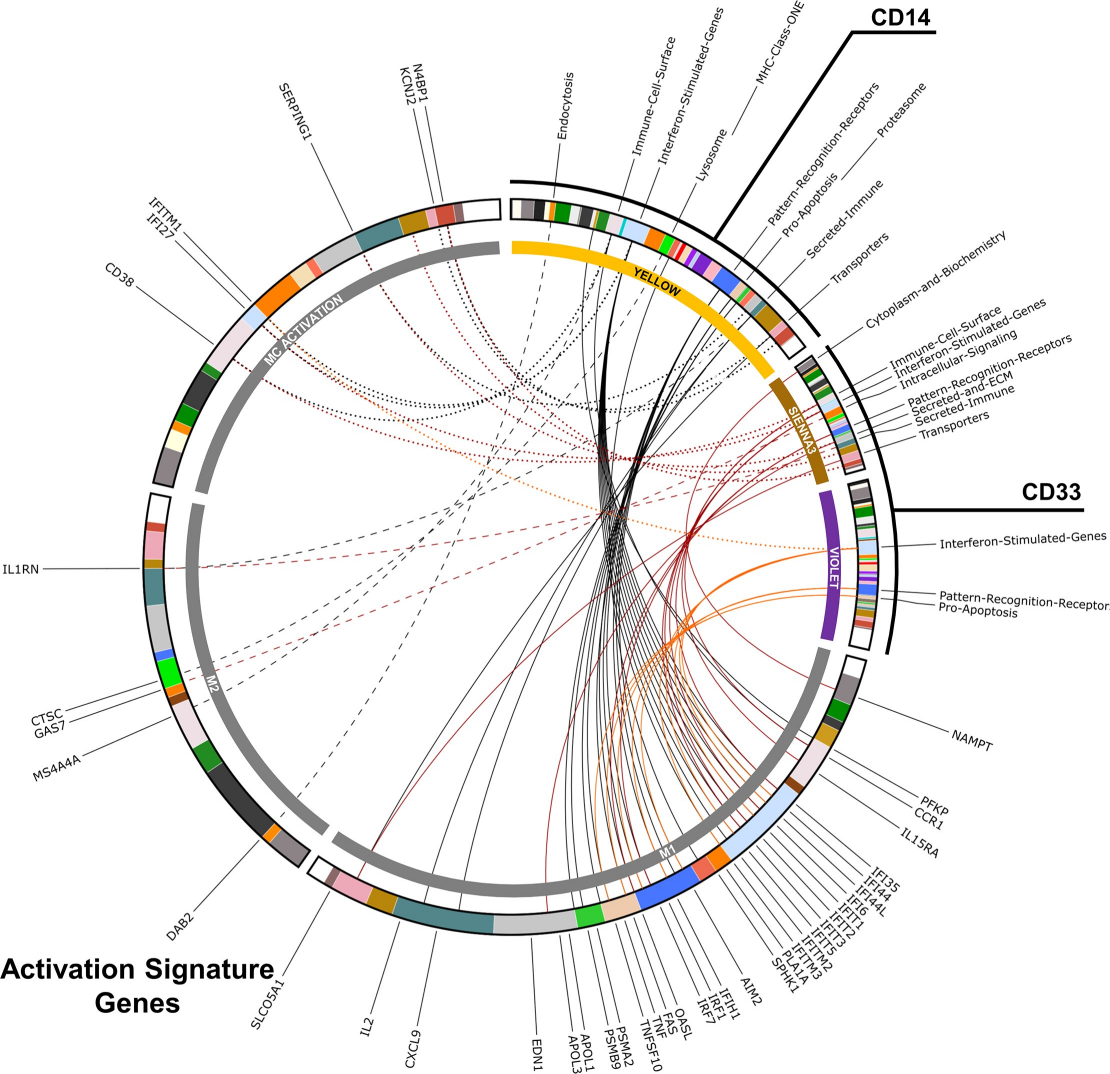
Activation signature genes found in disease-correlated WGCNA modules from active SLE MC. CIRCOS diagram comparing the composition of SLE positively-correlated CD14^+^ and CD33^+^ WGCNA modules to genes enriched in M1- or M2-polarized human Mϕ or genes associated with general MC activation (upregulated in both M1 and M2 conditions). Gene lists defining these signatures were adapted from Martinez et. al., 2006.[52] Genes found in the yellow module (CD14^+^) are shown in black, genes found in the violet module (CD33^+^) are shown in red, and genes found in the sienna3 module (CD33^+^) are shown in orange. M1-related genes are represented with solid lines, M2-related genes are represented by dashed lines, and general MC activation genes are represented with dotted lines.

In order to determine the gene signatures that were relevant to SLE pathogenesis in an unbiased manner, we generated gene expression modules via WGCNA with correlation to clinical traits, then prioritized those with correlation to disease cohort and even eigengene distribution to exclude modules whose assembly were driven primarily by a single eigengene. As the CD33^+^ dataset contained no inactive SLE patients, data from only active SLE patients was used to construct modules for comparison (CD14^+^ inactive results are shown in S1 Fig. The CD14^+^ dataset produced one module with significantly positive correlation to SLE (yellow: n = 362, r = 0.837, p = 4.22e-6) and one module with significantly negative correlation to SLE (sienna3: n = 229, r = -0.852, p = 1.84e-6), and the CD33^+^ dataset produced two modules significantly positively correlated to SLE (violet: n = 182, r = 0.718, p = 7.88e-4; sienna3: n = 133, r = 0.784, p = 1.17e-4) and one module significantly negatively correlated to SLE (darkolivegreen: n = 227, r = -0.549, p = 0.0182) (Fig 2B). Notably, the CD14^+^-derived modules were also significantly correlated to SLEDAI (r = 0.651, p = 1.88e-3 and r = -0.641, p = 2.31e-3 respectively). The significantly positive disease-correlated modules from the CD14^+^ dataset contained several activation-related genes, mostly concentrated in the apoptosis, ISG, and PRR BIG-C categories (visualized in Fig 3). While the yellow module was heavily enriched for M1-related genes, four M2-related genes were also present. Of the 37 genes in this module that were associated with MC activation, 27 (73%) were M1-related genes. The CD33^+^ modules each contained far fewer activation genes and almost no M2 signature. Despite this, of the 29 MC activation-associated genes in both these modules combined, 21 (72%) were M1 genes. The CD14^+^ negatively-correlated module (sienna3) contained no MC activation genes and the CD33^+^ negatively-correlated module (darkolivegreen) contained only one, GAS7 (S4 Table). These findings support the hypothesis that Mϕ activation state contributes heavily to the differential MC DE gene signature between active and inactive SLE. Furthermore, the polarization genes present are nearly exclusively M1-associated, suggesting that the observed differences in Mϕ polarization may be driving enhanced inflammation in active SLE.

### Protein interaction-based clustering of genes in WGCNA modules significantly correlated to disease activity

We then carried out a more detailed analysis of the composition of the WGCNA modules significantly correlated to disease activity by using Cytoscape with the stringApp and MCODE plugins to create protein-protein interaction networks and clusters. The resulting networks were further simplified into metastructures defined by the number of genes in each cluster, the number of significant intra-cluster connections identified by MCODE, and the strength of associations connecting members of different clusters to each other. This dual approach allowed us to compare the overall topology of different WGCNA clusters while also noting genes of interest and their groupings.

**Fig 4:**
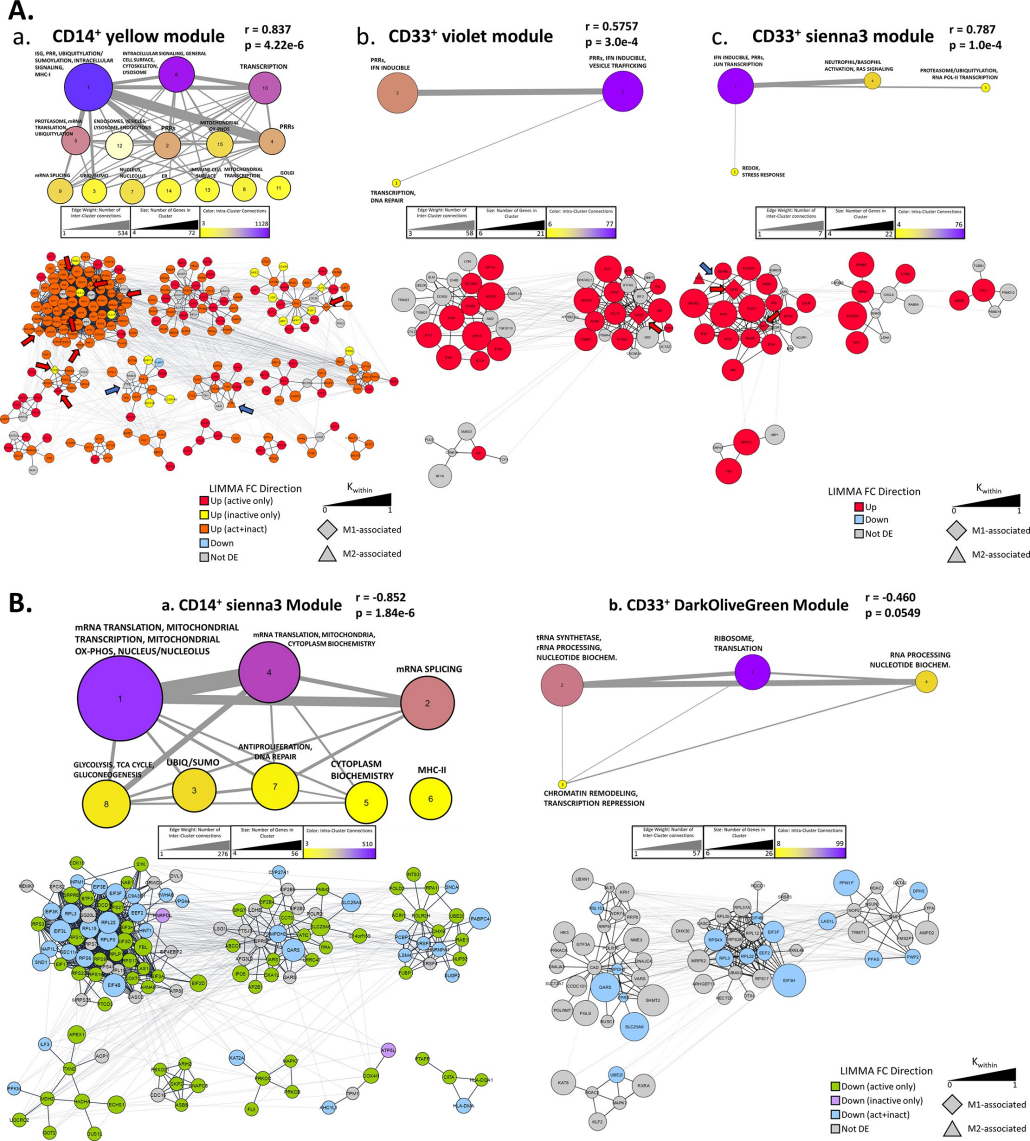
Protein interaction-based clustering of genes in WGCNA modules significantly correlated to SLEDAI. Protein-protein interaction networks and clusters generated via CytoScape using the STRING and MCODE plugins. Networks were constructed of the gene lists of WGCNA modules positively (A, above) or negatively (B, below) correlated to SLEDAI from CD14^+^ MC (a) or CD33^+^ MC (b, c). MCODE clusters are determined by the strength of protein-protein interactions, calculated by pooling information from publicly available literature. Top half of diagrams show the cluster metastructure of each network while bottom half shows the specific genes that make up each cluster. M1-related genes are indicated by red arrows and M2-related genes are indicated by blue arrows.

The largest and most internally connected cluster of genes in the CD14^+^ *yellow* module (positively correlated to disease activity, Fig 2B) was dominated by ISG and PRR-related genes and contained several members of the ubiquitin C pathway, a gene network not present in either of the positively correlated CD33^+^ modules (Fig 4A, top). Interestingly, further analysis of this cluster and the closely related proteasome/mRNA translation/ubiquitylation cluster revealed several upregulated activation-induced genes, including M1-associated genes (Fig 4Aa, bottom, red arrows). Two of the four M2-associated genes in the module (CTSC and IL1RN) appeared in smaller PRR and vesicle-associated clusters (Fig 4Aa, blue arrows). Similar PRR/vesicle clusters were found in the two positively correlated (Fig 2B) CD33^+^ modules, but only three M1 genes appeared in these clusters (Fig 4Ab and 4Ac; red arrows). Taken together, these data suggest that dysregulated activation signals in CD14^+^ MC drive SLE pathogenesis, especially in patients with active disease. The two WGCNA modules negatively correlated to SLEDAI (Fig 2B, *sienna3* for CD14^+^ and *darkolivegreen* for CD33^+^) were less informative and broadly mirrored each other in content, both containing networks related to RNA synthesis and processing, translation, and DNA maintenance (Fig 4B). Two clusters that arose from the CD14^+^ module represented pathways not present in the CD33^+^ module: glycolysis/TCA cycle/gluconeogenesis in cluster 8 and ubiquitylation/sumoylation in cluster 3. The majority of the genes in these clusters were selectively downregulated in active SLE only (Fig 4Ba).

### Predicted compounds targeting CD14^+^ MC pathways in SLE

With the goal of identifying novel potential therapies for SLE, DE gene data from CD14^+^ MC were used as input for LINCS, a drug discovery tool based upon gene expression changes induced by perturbagens in a variety of reference cell lines. The result is a list of drugs that counteract the genomic changes that propagate disease, determined in an unbiased manner and based on empirical data.

Summarized results of the LINCS analysis are presented in Tables 1 and 2 for the CD14^+^ MC obtained from active SLE patients and inactive SLE patients, respectively. Compounds directed against a shared target are collapsed into each category, allowing calculation of LINCS connectivity score statistics for all drugs impacting that target. The drug with the strongest connectivity score for each target is shown in the “Representative Drug” column. Notably, 49% of targets and 44% of representative drugs were suggested by LINCS for both active and inactive SLE MC (Tables 1 and 2, bolded). Cross-referencing the results against FDA and clinical trial databases reveals that many of the LINCS-suggested drugs are either already approved or in trials for non-lupus indications, underscoring their potential for swift and successful drug repositioning (Tables 1 and 2, indicated by ^†^ and ^‡^).

**Table 1:** Compounds targeting CD14^+^ monocyte pathways in active SLE.

Target	Count	Range	Mean ± SEM	Representative Drug
**Farnesyl transferase**	2	(-95.98)—(-99.61)	-97.79 ± 1.81	**Tipifarnib‡**
**Acetylcholinesterase**	2	(-93.16)—(-98.09)	-95.63 ± 2.47	Mestinon†
**PKC (pan)**	2	(-93.81)—(-97.19)	-95.50 ± 1.69	**bisindolylmaleimide-ix**
**mTORC1/2 (Tacrolimus^5^†)**	6	(-89.05)—(-99.66)	-94.70 ± 1.5	**KU-0063794**
**Sigma receptor**	2	(-86.80)—(-94.33)	-90.56 ± 3.76	**BD-1063**
**PI3K (pan) (Idelalisib^1^†)**	2	(-86.90)—(-93.15)	-90.02 ± 3.12	**GSK-1059615**
**ROCK-1/2 (KD025^7^‡)**	3	(-79.19)—(-95.90)	-89.96 ± 5.39	GSK-429286A
**PLK1**	2	(-87.31)—(-92.57)	-89.94 ± 2.63	ON-01910
**IGF-1R**	5	(-76.11)—(-99.20)	-89.87 ± 4.24	GSK-1904529A
**mTORC1 (Tacrolimus^5^†)**	3	(-85.83)—(-97.22)	-89.83 ± 3.7	**AZD-8055**
**HDM2**	3	(-81.37)—(-96.81)	-89.73 ± 4.5	HLI-373
**Ca channel**	9	(-82.26)—(-99.98)	-89.70 ± 2.45	**Nifedipine†**
**GR agonist**	12	(-74.94)—(-99.03)	-89.61 ± 2.18	**Dexamethasone†** ^**T**^
**CDK1, 2, 5 (Palbociclib^4^†)**	2	(-88.47)—(-89.62)	-89.05 ± 0.58	Aloisine
**PI3Kg**	2	(-81.84)—(-96.21)	-89.03 ± 7.19	**AS-605240**
**DNA-PK**	2	(-88.33)—(-88.67)	-88.50 ± 0.17	**NU-7026**
**MAP2K1/2**	6	(-78.58)—(-97.06)	-88.42 ± 2.9	**U0126**
**MAPK**	5	(-81.41)—(-92.96)	-88.39 ± 2.18	EO-1428
**Tyrosine Kinase (broad)**	4	(-82.14)—(-98.80)	-88.30 ± 3.83	**Lestaurtinib‡**
**PARP-1 (Niraparib^3^†)**	5	(-79.37)—(-91.68)	-87.95 ± 2.38	Rucaparib‡
**PDGFR**	2	(-87.63)—(-88.20)	-87.91 ± 0.28	tyrphostin-AG-1295
**JNK (pan)**	2	(-82.84)—(-92.78)	-87.81 ± 4.97	AS-601245
**EGFR (Gefitinib^1^†)**	10	(-74.73)—(-99.36)	-87.27 ± 2.99	**Lapatinib**^**0**^**†**
**b2 adrenergic receptor agonist**	6	(-80.38)—(-93.93)	-87.27 ± 2.23	Formoterol†
**5-HT 1B agonist**	3	(-83.27)—(-89.31)	-86.94 ± 1.86	Anpirtoline
**topoisomerase I (Irinotecan^-1^†)**	2	(-82.61)—(-90.87)	-86.74 ± 4.13	**Topotecan†**
**topoisomerase II**	3	(-81.58)—(-90.76)	-86.55 ± 2.68	**Razoxane†**
**Proton pump**	2	(-85.41)—(-87.66)	-86.53 ± 1.13	**Rabeprazole†**
**NMPRTase**	3	(-77.90)—(-94.32)	-85.94 ± 4.74	APO-866
**Enkephalinase**	2	(-84.66)—(-86.33)	-85.49 ± 0.83	Thiorphan‡
**Angiotensin II receptor**	2	(-84.60)—(-85.32)	-84.96 ± 0.36	Telmisartan†
**Aurora kinase A**	2	(-84.83)—(-84.87)	-84.85 ± 0.02	**MLN-8054‡**
**PI3Kb**	3	(-80.29)—(-88.89)	-84.77 ± 2.49	TGX-221
**K channel**	3	(-78.57)—(-87.81)	-84.42 ± 2.94	Paxilline
**b3 adrenergic receptor agonist**	3	(-77.43)—(-91.37)	-83.95 ± 4.05	L-755507
**PDE4 (Roflumilast^6^†)**	2	(-79.38)—(-87.35)	-83.36 ± 3.98	Ibudilast‡
**HMG-CoA reductase (Statins^3^†)**	6	(-76.34)—(-95.08)	-83.19 ± 3.05	**Atorvastatin†** ^**T**^
**ER (pan) (Tamoxifen^2^†)**	3	(-75.46)—(-87.80)	-82.61 ± 3.70	**Clomifene‡**
**VEGFR2 (Sorafenib^-3^‡)**	2	(-75.75)—(-86.67)	-81.21 ± 5.46	Orantinib‡
**Na channel**	2	(-75.75)—(-85.04)	-80.39 ± 4.64	**Benzamil**
**ATM Kinase**	2	(-78.69)—(-80.42)	-79.55 ± 0.87	CP466722
**AMPA receptor**	2	(-77.82)—(-80.21)	-79.02 ± 1.20	GYKI-52466
**Wnt (pan)**	2	(-76.35)—(-80.74)	-78.55 ± 2.20	PNU-74654
**HSP90**	2	(-76.64)—(-79.99)	-78.32 ± 1.68	Gedunin
**SERT**	2	(-75.00)—(-75.31)	-75.16 ± 0.15	Duloxetine†

†: FDA-approved compounds

‡: drugs in clinical trials or drugs in development

T: known utility in lupus therapy

Where applicable, CoLTS scores (range -16 to +11) are displayed as integers in superscript.[39]

**Table 2:** Compounds targeting CD14^+^ monocyte pathways in inactive SLE.

Target	Count	Range	Mean ± SEM	Top Drug
**PKC (pan)**	2	(-98.19)—(-99.03)	-98.61 ± 0.42	**bisindolylmaleimide-ix**
**IGF-1R**	4	(-93.01)—(-98.13)	-96.56 ± 1.21	BMS-536924
**mTORC1/2 (Tacrolimus^5^†)**	6	(-90.39)—(-99.97)	-96.00 ± 1.72	**KU-0063794**
**Farnesyl transferase**	2	(-94.89)—(-96.95)	-95.92 ± 1.03	**Tipifarnib‡**
**PI3K (pan) (Idelalisib^1^†)**	2	(-90.28)—(-99.74)	-95.01 ± 4.73	**GSK-1059615**
**topoisomerase I (Irinotecan^-1^†)**	3	(-90.33)—(-97.63)	-94.70 ± 2.23	**Topotecan†**
**mTORC1 (Tacrolimus^5^†)**	4	(-86.57)—(-98.97)	-94.29 ± 2.87	**AZD-8055**
**HDM2**	3	(-87.19)—(-96.91)	-93.25 ± 3.05	JNJ-26854165
**B-Raf**	2	(-89.18)—(-97.19)	-93.19 ± 4.01	Vemurafenib^-6^†
**FAAH**	2	(-90.89)—(-95.32)	-93.10 ± 2.22	PF-3845
**ROCK-1/2 (KD025^7^‡)**	2	(-91.66)—(-93.36)	-92.51 ± 0.85	Y-27632
**PI3Kb (Idelalisib^1^†)**	3	(-82.45)—(-98.27)	-92.34 ± 4.98	PI-828
**MAP2K1/2**	6	(-87.89)—(-98.76)	-92.17 ± 1.57	**U0126**
**DNA-PK**	2	(-85.41)—(-97.43)	-91.42 ± 6.01	**NU-7026**
**PI3Kg**	2	(-87.49)—(-94.95)	-91.22 ± 3.73	**AS-605240**
**TRPV1**	2	(-88.65)—(-92.83)	-90.74 ± 2.09	Eriodictyol‡
**COX-1**	2	(-88.36)—(-92.43)	-90.40 ± 2.03	eicosatetraynoic-acid
**PARP-1 (Niraparib^3^†)**	2	(-89.49)—(-91.14)	-90.31 ± 0.83	Olaparib^0^†
**HMG-CoA reductase (Statins^3^†)**	7	(-79.06)—(-97.06)	-89.27 ± 2.37	**Atorvastatin†** ^**T**^
**NK1**	2	(-79.34)—(-97.98)	-88.66 ± 9.32	FK-888
**Syk**	2	(-81.58)—(-94.43)	-88.01 ± 6.43	Fostamatinib^7^‡
**5-HT 1B agonist**	2	(-85.72)—(-90.07)	-87.90 ± 2.18	5-nonyloxytryptamine
**NMPRTase**	2	(-82.20)—(-92.79)	-87.50 ± 5.30	CAY-10618
**Sigma receptor**	2	(-86.24)—(-88.50)	-87.37 ± 1.13	**BD-1063**
**Ca channel**	11	(-76.85)—(-99.83)	-87.01 ± 2.48	**Nifedipine†**
**Adrenergic receptor (pan) agonist**	2	(-76.15)—(-97.26)	-86.71 ± 10.56	Dopamine†
**CB2 agonist**	3	(-82.69)—(-92.44)	-86.51 ± 3.01	GW-405833
**SERT**	4	(-76.46)—(-93.45)	-85.91 ± 4.03	Paroxetine†
**topoisomerase II**	5	(-77.70)—(-94.56)	-85.78 ± 2.90	**Razoxane†**
**EGFR (Gefitinib^1^†)**	16	(-74.54)—(-99.27)	-85.32 ± 2.00	**Lapatinib**^**0**^**†**
**Tyrosine kinase (broad)**	4	(-79.11)—(-99.44)	-85.16 ± 4.79	**lestaurtinib‡**
**MAPK**	2	(-74.80)—(-95.28)	-85.04 ± 10.24	JX-401
**5-HT 4**	2	(-81.68)—(-87.92)	-84.80 ± 3.12	RS-23597-190
**ER (pan)**	6	(-75.38)—(-96.20)	-84.18 ± 2.99	**Clomifene‡**
**VEGFR (pan) (Sorafenib^-3^‡)**	3	(-77.91)—(-87.26)	-83.40 ± 2.82	Tivozanib‡
**BCL-2 (Venetoclax^0^†)**	2	(-77.03)—(-89.71)	-83.37 ± 6.34	ABT-737‡
**ATM Kinase**	2	(-75.51)—(-89.82)	-82.67 ± 7.15	KU-55933
**c-Met**	2	(-77.20)—(-88.11)	-82.65 ± 5.46	SU-11274
**Proton pump**	2	(-78.63)—(-84.47)	-81.55 ± 2.92	**Rabeprazole†**
**GR agonist**	3	(-75.23)—(-89.53)	-81.19 ± 4.30	**Dexamethasone†** ^**T**^
**H3 receptor**	2	(-76.33)—(-85.49)	-80.91 ± 4.58	iodophenpropit
**Aurora kinase A**	2	(-78.64)—(-81.87)	-80.26 ± 1.61	**MLN-8054‡**
**b3 adrenergic receptor agonist**	2	(-77.27)—(-82.22)	-79.74 ± 2.47	SR-59230A
**VEGFR2 (Sorafenib^-3^‡)**	2	(-76.31)—(-81.30)	-78.81 ± 2.49	SU-4312
**b2 adrenergic receptor agonist**	3	(-77.03)—(-78.85)	-78.00 ± 0.53	Fenoterol‡
**Na channel**	2	(-76.38)—(-79.17)	-77.78 ± 1.40	**benzamil**

†: FDA-approved compounds

‡: drugs in clinical trials or drugs in development

T: known utility in lupus therapy.

Where applicable, CoLTS scores (range -16 to +11) are displayed as integers in superscript.[39]

### Projected upstream regulator genes in CD14^+^ MC isolated from active and inactive SLE patients

**Fig 5:**
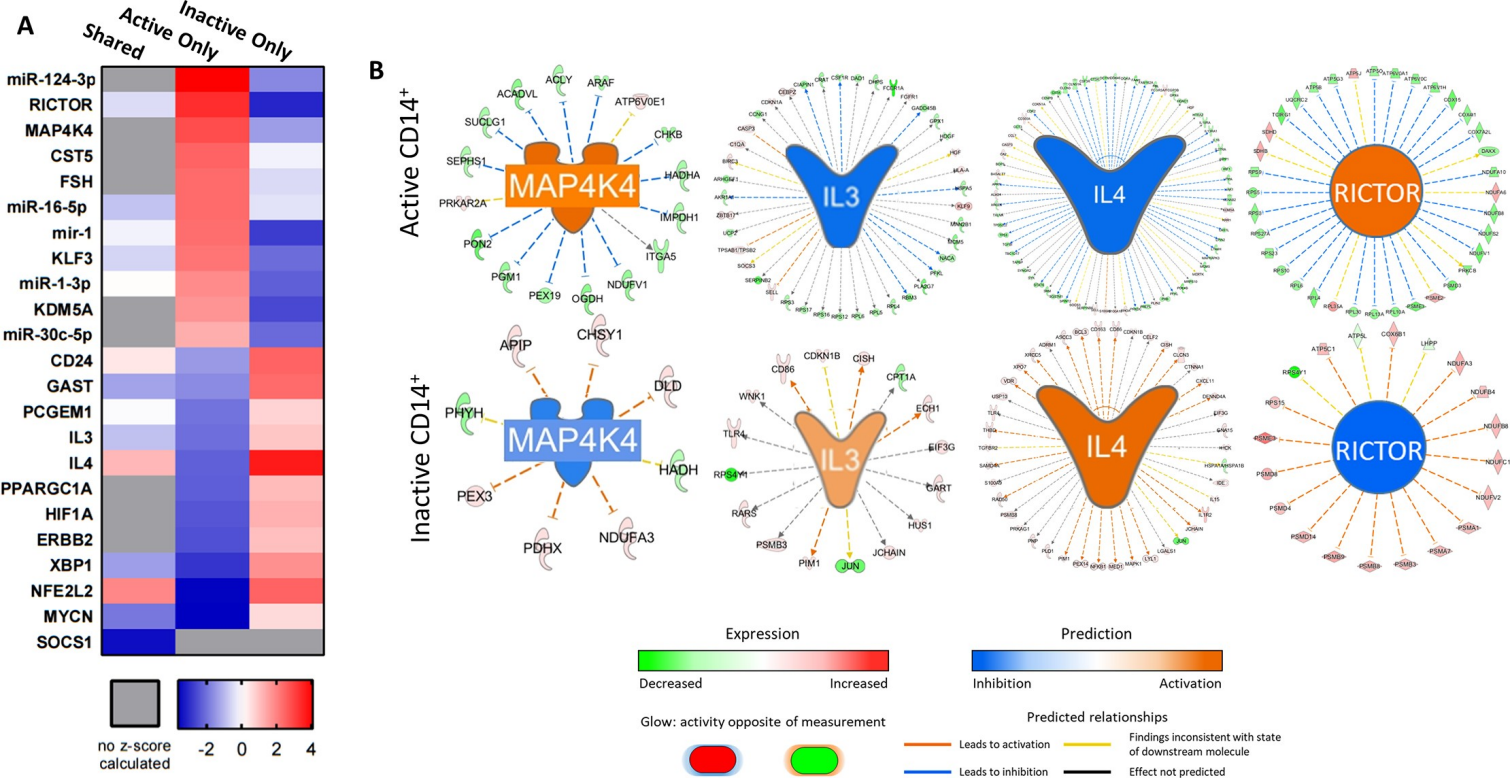
Projected upstream regulator genes in CD14^+^ monocytes isolated from active and inactive SLE patients. (A) IPA was used to analyze the CD14^+^ MC dataset and identify putative upstream regulators for active patient monocytes, inactive patient monocytes, and the active-inactive overlap using a p-value cutoff of 0.05. Only genes for which IPA assigned a z-score of ≥|2| in at least one of the three sets are shown. (B) Representative diagrams showing downstream gene expression changes (outer circles) used to calculate upstream regulators (center).

To investigate the intracellular signaling pathways at play, we employed IPA to analyze the CD14^+^ MC DE dataset and identify potential biologic upstream regulators (BURs) for MC from active patients, inactive patients, and the active-inactive overlap (Fig 5A). Genes for which IPA indicated a z-score of ≥ 2 in at least one of the three sets are shown. Several of the resulting genes are known to be major regulators of MC polarization, including the M1 regulators MAP4K4 and mir-1 and the M2 regulators IL3, IL4, PPARGC1A, HIF1A, and NFE2L2 (Fig 5A). Notably, the z-scores show a clear delineation of their opposing activities in active SLE patient MC vs inactive SLE patient MC, with M1 regulators displaying positive z-scores in active patients and negative z-scores in inactive patients and vice-versa for M2 regulators. Each of these trends was supported by the corresponding expression of several downstream genes known to interact with each upstream regulator (Fig 5B). Interestingly, only one gene known to be involved in Mϕ polarization had a z-score that contradicted this pattern: RICTOR, a relative of mTOR and a subunit of the mTORC2 complex, has been shown to suppress M1 polarization in mice yet is identified by IPA as an upstream regulator of CD14^+^ MC from active SLE patients.[55]

**Fig 6:**
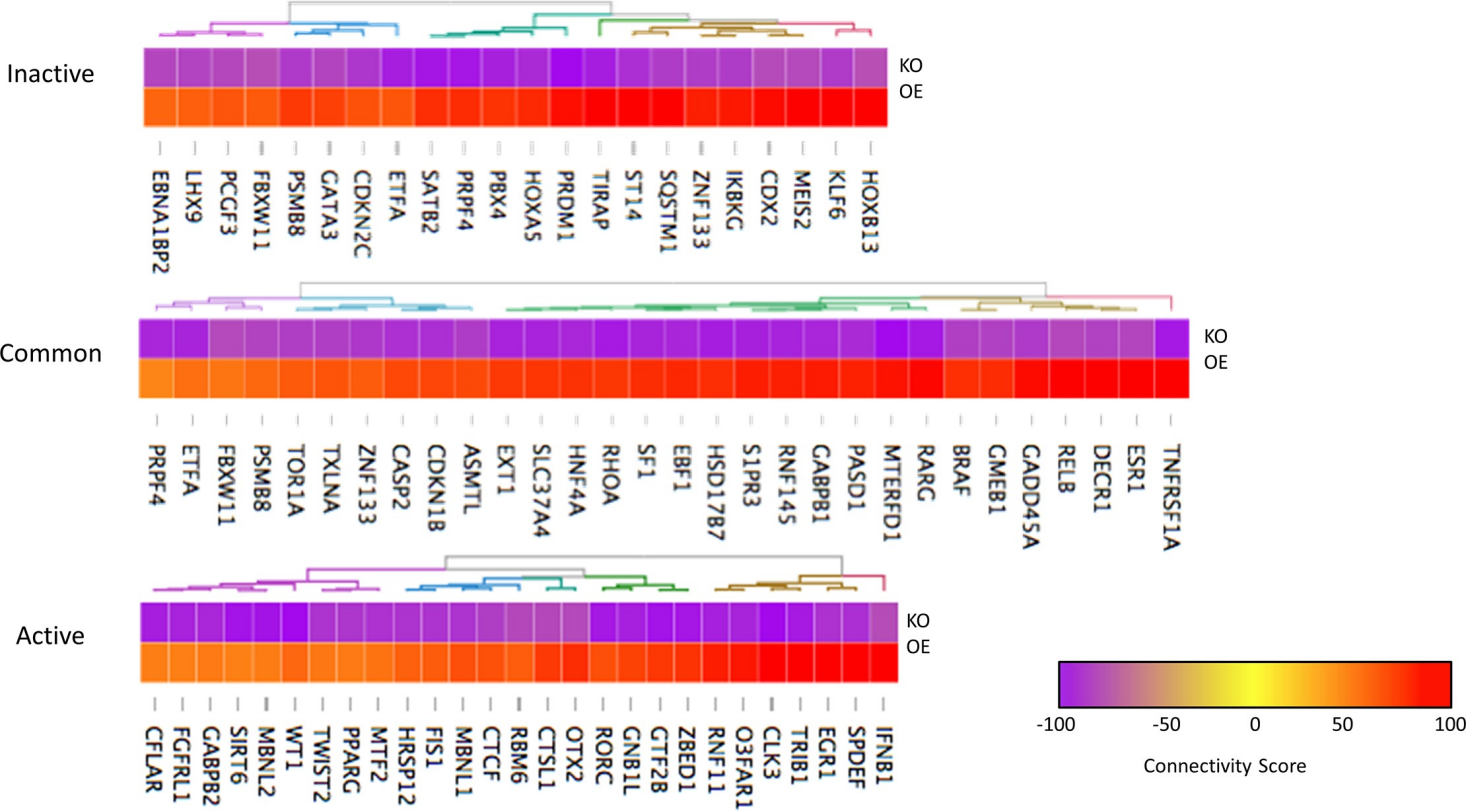
LINCS biological upstream regulators. Gene sets from CD14^+^ MC isolated from active or inactive SLE patients were used as input for the LINCS analysis platform, which reports connectivity scores for individual genes that describe how well the genomic change between the baseline and experimental input sets matches the change observed following the knockdown or overexpression of the individual gene in question. Knockdown and overexpression data were filtered by genes for which LINCS reported connectivity scores for both categories, and genes were identified as BURs for a particular dataset if they received a knockdown connectivity score between -75 and -100 and an overexpression connectivity score between 50 and 100 for that dataset.

We also sought to utilize the gene connectivity scores from the collection of knockdown and overexpression experiments present in the LINCS database to identify BURs determined primarily by empirical results. Genes were identified as BURs for a particular dataset if they received a knockdown connectivity score between -75 and -100 and an overexpression connectivity score between 50 and 100 for that dataset. This approach produced 17 BURs unique to the inactive SLE cohort, 31 BURs unique to the active SLE cohort, and 30 BURs common to both (Fig 6). These regulators were distinct from those identified by IPA, representing additional potential drug targets.

### Machine learning confirms that gene modules from MC predict SLE activity in unrelated data sets

The relationships between MC gene expression and SLE activity suggested that a machine learning method might be able to predict disease activity when “trained” with MC gene signatures. To this end, unrelated WB and PBMC datasets were merged into a test set and analyzed for MC WGCNA module enrichment via GSVA. In order to compare the predictive power of MC gene signatures, WGCNA modules were also generated for CD4 T cells, CD19 B cells, plasma cells (PC) and low-density granulocytes (LDG) and employed in a similar manner to predict disease activity.

**Fig 7:**
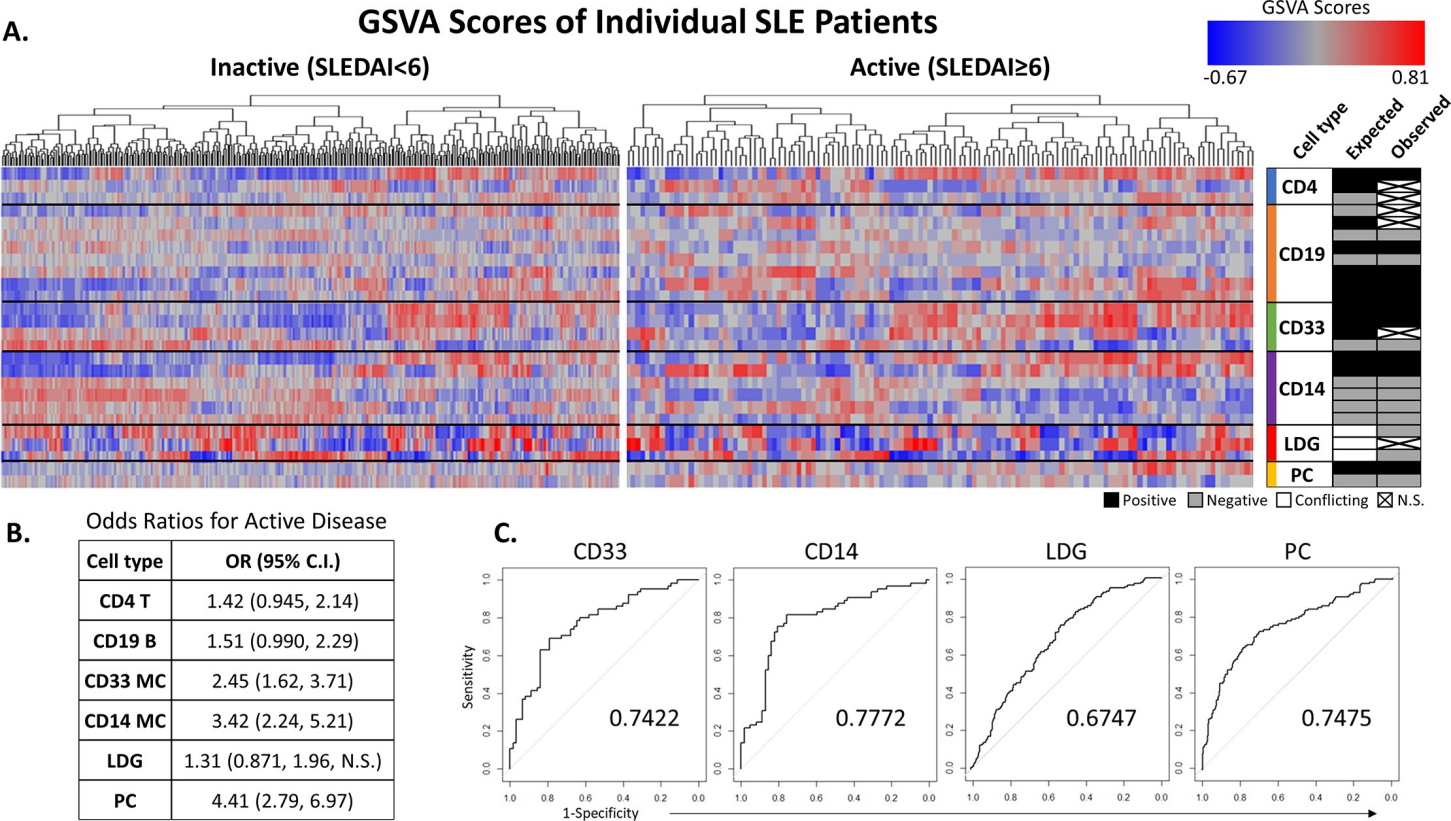
Cellular gene signature modules provide basis for machine learning predictions of SLE activity. (A) GSVA was utilized to generate scores to assess enrichment of WGCNA lymphocyte subset gene modules correlated with disease activity in WB or PBMC samples separated into inactive or active SLE patients. Results are shown following unsupervised hierarchical clustering. The expected and observed correlations to disease states of each module and the cell type of their origin are shown on the right (black: positive correlation; gray: negative correlation; white: unknown correlation; x: no significant correlation). (B) Odds ratios (OR) with 95% confidence intervals (CI) were calculated from the GSVA data to determine the strength of association of each cellular module with active disease. (C) ROC curves displaying representative results of disease activity prediction by the generalized linear model algorithm for modules from an individual cell type. Area under the curve is shown on each panel.

Hierarchical clustering of GSVA scores indicated that enrichment of some modules (PC, CD14+ MC) was more frequently observed in active compared to inactive SLE, although complete separation of active versus inactive samples was not achieved. To explore this in greater detail, odds ratios (OR) for the likelihood of the enrichment of various WGCNA modules from different cell types in active SLE were calculated by comparing the expected versus observed enrichment of each module. As expected (since increased PC are associated with disease activity [51]), PC modules manifested the highest OR for active disease at 4.41, whereas LDG modules exhibited the lowest OR (1.32), consistent with the previous observation that increases in LDG activity do not correlate with disease activity in SLE [50] (Fig 7A and 7B). Notably, MC modules outperformed either CD4 T cells (OR: 1.42) and CD19 B cells (1.51), with CD14^+^ MC exhibiting a higher OR (3.42 vs 2.45). GSVA scores were then used as input for a Generalized Linear Model-based machine learning algorithm which attempted to identify whether samples from the WB and PBMC test set were obtained from active or inactive SLE patients. CD33 and CD14 MC signatures surpassed LDG signatures and performed at least as well as PC signatures in accuracy as measured by the area under the resulting ROC curves (Fig 7C).

## Discussion

Here we describe a comprehensive, bioinformatic approach to identify cell type-specific patterns of genetic variation among active and inactive SLE patients and to identify high-priority candidate compounds for drug repositioning efforts. Whereas bioinformatic analysis is often used to supplement studies of SLE pathogenesis in murine models or *in vitro*, our work represents a novel “big data” strategy of applying these techniques to patient-derived data in order to identify constellations of genes that might determine clinical outcomes in specific patients.

Our initial findings that MC expressed a considerable number of DE genes in both active and inactive patients compared to healthy controls whereas B and T cells only expressed a significant DE gene signature in active patients compared to healthy controls led us to hypothesize a critical role for MC in human SLE in agreement with previous studies in lupus-prone mice. B and T cell activity along with that of MC contribute to disease activity in SLE, whereas the altered genomic signatures of MC might preserve the disease state of inactive SLE between flares and could even affect the transition between active and inactive SLE.

Our analyses of M1 and M2 signatures indicate that although there is overlap, M2 gene expression is more prominent in inactive SLE patients whereas M1 gene expression is highly enhanced in active SLE patients. This confirms several recent studies that have investigated the roles of Mϕ polarization and DC activation in SLE-like conditions: overabundance of proinflammatory M1 Mϕ and decreased expression of the M2 marker CD206 were detected in both lupus-prone mice and SLE patients [56,57], and therapeutic stimulation of M2 polarization significantly decreased disease severity in an induced murine SLE model.[33] However, experimental intervention in M2b polarization as well as microRNA array profiling suggest that M2b Mϕ may contribute to SLE severity, indicating that the relationship between Mϕ polarization and lupus progression is more nuanced than it appears at first glance.[57,58]

Use of GSVA to compare expression patterns against our BIG-C database revealed differences in upregulated pathways in MC derived from active and inactive SLE patients that mirror and reinforce the M1/M2 signatures observed in the DE genes. As expected in SLE, MC from both active and inactive patients are enriched for categories related to IFN signaling and inflammation compared to healthy controls. In contrast, MC from active patients uniquely downregulate pathways related to mitochondrial function and glycolysis in favor of immune cell surface markers and secreted factors while MC from inactive patients downregulate genes in the cell surface category and are enriched for ubiquitination and sumoylation pathways. These data suggest that MC from active SLE patients favor pro-inflammatory M1-related pathways while MC from inactive patients favor M2-related pathways involved in resolution of the immune response.

Upstream regulator analysis using IPA further confirmed this conclusion, identifying several M2-associated factors as positive regulators in MC from inactive SLE patients but not active patients, including IL-3, IL-4, and HIF1A (Fig 5). Interestingly, the upstream regulator with the strongest differential z-score preference for active MC versus inactive MC was also the only M2 gene identified as an exclusive regulator for active patient MC: RICTOR, an mTORC2 component RICTOR previously shown to inhibit M1 polarization. This result may simply reflect an expected component of the elevated inflammatory profile of an SLE patient compared to a healthy patient or it may suggest a specific role for RICTOR and the mTORC2 complex in the transition between inactive and active SLE. Further study is necessary to make this distinction.

Attempting to identify biological upstream regulators empirically by matching gene knockdown and overexpression results from the LINCS analysis platform, on the other hand, revealed practically no polarization-related genes despite identifying several regulators unique to the inactive or active cohorts (Fig 6). Despite this, these results greatly expanded the potential list of upstream regulators and may suggest pathways with a unique and yet undocumented role in macrophage polarization. Furthermore, these findings extend to the targets and compounds predicted to be useful by LINCS in reverting the gene signatures of active or inactive SLE patients back to the baseline of healthy controls (Tables 1 and 2). Although unique targets and compounds were identified for active and inactive SLE patients, these did not follow a clear pattern of M1- or M2-related inhibitors. This, along with the lack of polarization genes among LINCS BURs, may in part be related to the inception of the LINCS project as a search for cancer treatments, resulting in a preference for antiproliferative drugs and a higher sensitivity to genes that control proinflammatory signaling pathways. Nonetheless, the presence of both shared and unique targets suggests that this approach can be used either to identify drugs with the potential to treat the SLE signature as a whole or to find therapies tailored toward the presentation of an individual patient’s disease. The novel drugs and targets resulting from this analysis will need to be individually evaluated, screened, and tested to confirm efficacy in SLE treatment.

One limitation of these analyses, however, was that they were all performed within the same two GEO datasets (GSE10325 and GSE38351). As a result, overlapping findings had somewhat limited value for the purposes of validation. The results obtained from ML analysis, therefore, presented two critical insights. First, ML findings confirm those in the literature that while PC genomic signatures correlate with disease activity, LDG genetic signatures do not (Fig 7B).[50,51] Second, the construction of a test set from GEO datasets unrelated to our initial analyses allowed for our ML approach to act as an impartial, external validation of our previous findings and conclusions regarding the impact of MC populations on SLE initiation and pathogenesis. Together, these confirmatory results validate the use of ML as a predictive (and potentially diagnostic) tool in SLE research and treatment.

Despite the prevalence of SLE and the considerable amount of research studying the link between gene expression and SLE activity [46,59,60], there is no definitive diagnostic tool available to determine either whether a patient has SLE or whether/when a patient will experience a flare. Extreme variation among SLE patients further complicates the issue: unsupervised hierarchical clustering of GSVA enrichment scores for disease-associated WGCNA modules produced no uniform pattern of association with SLE activity, and when performed again on pre-sorted datasets, each produced a small subgroup of patients whose enrichment highly resembled that of the other (Fig 7A). These overlapping groups were initially hypothesized to represent patients with intermediate SLEDAI scores in the process of transitioning between active and inactive disease, but this did not turn out to be the case, highlighting the degree of patient heterogeneity present in the test set and the need for further development of computationally intensive, multivariate analysis methods. Data presented here from our initial attempt to integrate our datasets into a predictive ML algorithm suggest that MC-derived gene signatures could predict disease activity as reliably as PC signatures which, unlike LDGs, have been shown to correlate with disease activity (Fig 7B and 7C).[50,51] These early MC signatures may provide the basis of a tool to diagnose SLE in its early stages (before PC expansion) or to detect alterations in MC that precede a flare. However, it is important to note that these signatures were derived from a relatively small number of SLE patients. Subsequent experiments, therefore, should refine and expand the ML approach to include MC samples from a larger cohort of patients.

## Conclusion

MC genomic signatures correlate with and successfully predict SLE disease activity. Whereas B and T cells only manifested DE genes in active SLE patients, DE genes could be detected in MCs from patients with both active and inactive SLE when compared to healthy controls. Examination of these signatures by multiple approaches confirmed the involvement of previously reported pathways (IFN signaling, inflammation, TLR/DAMP signaling) and also identified MC polarization-related pathways and genes as correlated with SLE activity. When used as input for an ML-based prediction algorithm, these MC-derived signatures were able to successfully predict active versus inactive SLE patient samples and did so more effectively than signatures from CD19 B cells and CD4 T cells. The predictive power of these MC signatures makes them compelling input data for perturbagen databases, enabling identification of promising novel and personalized treatment options for SLE.

## Supporting information

S1 Table Summary of datasets used for meta-analyses. (XLSX)

S2 Table Differentially expressed polarization genes in SLE CD14^+^ MC. M1- and M2-associated polarization genes found in LIMMA DE analysis of CD14^+^ MC in active and inactive SLE.
(XLSX)

S3 Table CD14^+^ MC DE BIG-C categories. Breakdown of genes from active and inactive CD14^+^ LIMMA DE analysis and their matching functional category from BIG-C annotation.
(XLSX)

S4 Table Composition of negatively-correlated MC WGCNA modules. (XLSX)

S1 Fig CD33^+^ MC WGCNA summary. (A) Dendrogram of modules generated by WGCNA of CD33^+^ MC and (B) their correlations to clinical traits.
(TIF)

S1 File FDAPBMC3 dataset. Log fold change expression values of PBMCs from SLE patients. Previously unpublished dataset obtained through collaboration with Arasappan et al. [49].
(ZIP)
