# Supplementary figures and images for: Identification of alterations in macrophage activation associated with disease activity in systemic lupus erythematosus
Source: PLoS One. 2018 Dec 18;13(12):e0208132. doi: 10.1371/journal.pone.0208132 (PMC6298676; doi:10.1371/journal.pone.0208132)

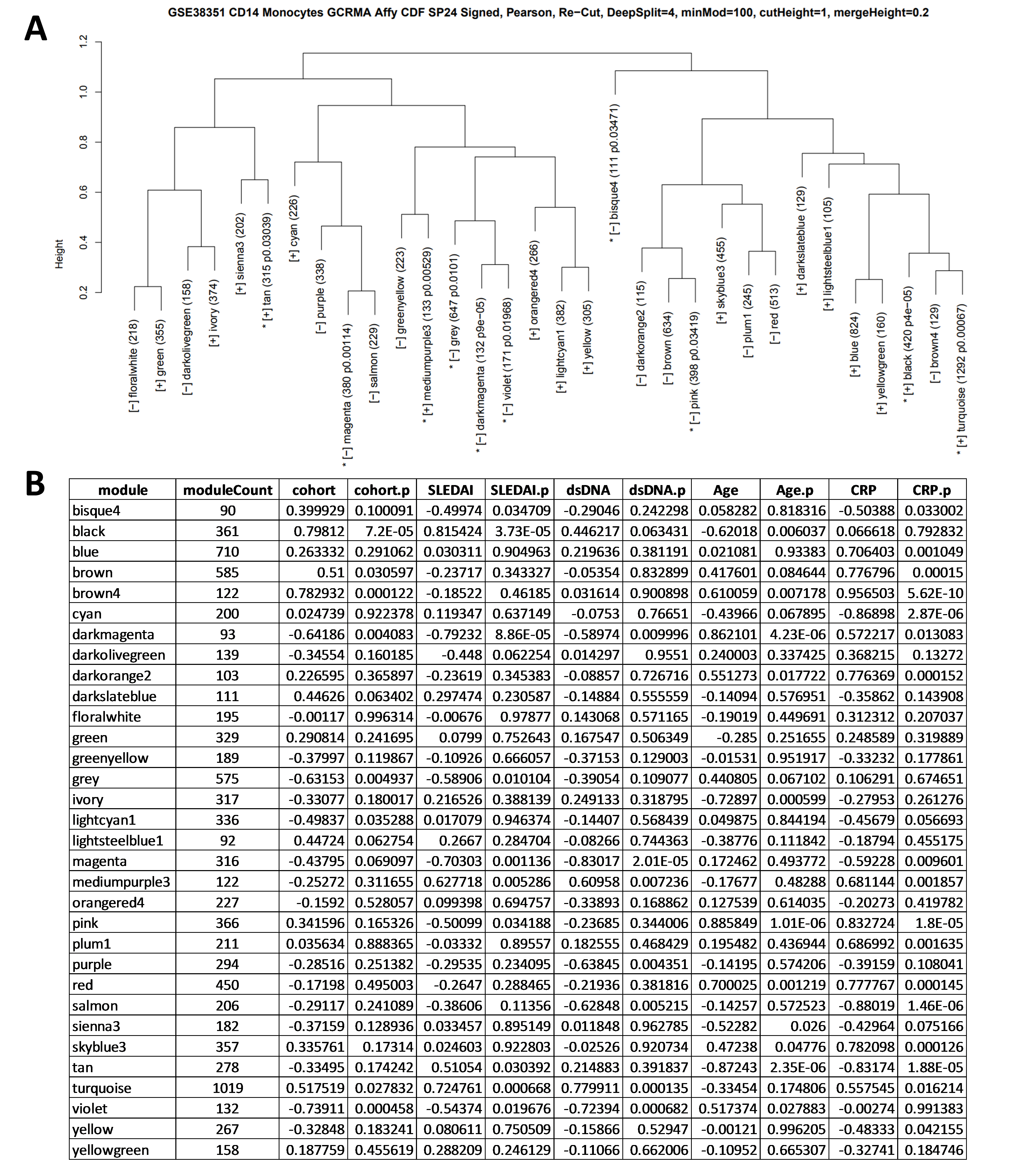

Supplement: S1 Fig — (A) Dendrogram of modules generated by WGCNA of CD33+ MC and (B) their correlations to clinical traits. (TIF) [file pone.0208132.s005.tif]
